# Tumor necrosis factor receptor 2/AKT and ERK signaling pathways contribute to the switch from fibroblasts to CAFs by progranulin in microenvironment of colorectal cancer

**DOI:** 10.18632/oncotarget.15461

**Published:** 2017-02-17

**Authors:** Linlin Wang, Dong Yang, Jing Tian, Aiqin Gao, Yihang Shen, Xia Ren, Xia Li, Guosheng Jiang, Taotao Dong

**Affiliations:** ^1^ Department of Radiation Oncology, Shandong Cancer Hospital and Institute, Shandong University, Jinan, Shandong 250117, P. R. China; ^2^ Department of Oncology, Jinan Central Hospital, Shandong University, Jinan, Shandong 250013, P.R.China; ^3^ Department of Oncology, Affiliated hospital of Jining Medical College, Jining, Shandong 272129, P. R. China; ^4^ Department of Oncology, People's Hospital of Zhangqiu City, Zhangqiu, Shandong 250200, P. R. China; ^5^ Programs of Cancer Biology, University of Hawaii Cancer Center, University of Hawaii, Honolulu, HI 96813, USA; ^6^ Key Medical Laboratory for Tumor Immunology and Traditional Chinese Medicine Immunology, Key Laboratory for Rare and Uncommon Diseases of Shandong, Institute of Basic Medicine, Shandong Academy of Medical Sciences, Jinan, Shandong 250012, P. R. China; ^7^ Department of Gynecology and Obstetrics, Qilu Hospital of Shandong University, Jinan, Shandong 250012, P. R. China

**Keywords:** PGRN, colorectal cancer, cancer associated fibroblasts, TNFR2, AKT

## Abstract

Cancer associated fibroblasts (CAFs) are a crucial cellular component in tumor microenvironment and could promote tumor progression. CAFs are usually derived from resident fibroblasts, which undergoing an activated process stimulated by tumor cells. However, the agents and mechanism driving this switch have not yet been elucidated. Progranulin (PGRN), a well acknowledged secreted glycoprotein, could promote proliferation and angiogenesis of colorectal cancer (CRC) cells, and high expression of PGRN correlated with patient poor prognosis. Whether PGRN has effects on the function of stromal fibroblasts is unknown. Herein we found that there was a positive correlation between PGRN expression of CRC cells and expressions of smooth muscle actin α (α-SMA) on CAFs in CRC patient tissues. PGRN/α-SMA co-expression was positively correlated with CRC patient poor prognosis. Co-cultured with CRC cells or human recombinant PGRN (rPGRN), the expression of Ki67, fibroblast activation protein (FAP) and α-SMA in fibroblasts were all up-regulated significantly, accompanying with elevated cellular proliferation, migration and contraction. Whilst co-cultured with PGRN-silenced CRC cells, these functions were down-regulated. Studies of the underlying molecular mechanism demonstrated that either tumor necrosis factor receptor 2 (TNFR2)/Akt or the extracellular regulated kinase (ERK) signaling pathway contributed to modulate of Ki67, FAP, and α-SMA expression, and correlated to abilities of proliferation, migration and contraction in fibroblasts. In conclusion, PGRN plays an important role in activation of CRC fibroblasts, which may be taken as a prospective target of CRC therapy.

## INTRODUCTION

As one of the most common malignant tumors in the world, the mortality of colorectal cancer (CRC) has declined in recent years, greatly attributing to early screening program and ameliorated therapy regimens, such as neoadjuvant chemotherapy, molecular target therapy, biotherapy, and so on. But for patients at advanced clinical stage, prognosis is still very poor [1–3]. A detailed understanding of molecular mechanisms regulating the development, progression, and metastasis of a malignant colorectal tumor may lead to improvements in patient therapy outcome.

It has been reported that a reactive microenvironment played important roles in tumor occurrence and progression [4, 5]. Cancer associated fibroblasts (CAFs) are one kind of the main cellular components in tumor microenvironment, and play important role in proliferation, migration and invasion of tumor cells through inducing epithelial mesenchymal transformation (EMT), secreting matrix metalloproteinase (MMP) or growth factors [4, 6–10]. It has been demonstrated that so far resident fibroblasts were considered as the main source of CAFs [5]. Stimulated by adjacent tumor cells, resident fibroblasts became more proliferative, aggressive and contractive and could transformed into CAFs, which expressed fibroblast activation protein (FAP) or smooth muscle actin α (α-SMA) [11–13]. But the mechanism of transformation from resident fibroblasts to CAFs is still elusive.

Progranulin (PGRN) is a secreted glycoprotein composed of 7.5 repeats of cysteine-rich motif [14–18], and has been reported to play important role in cancer progression and prognosis through promoting proliferation, drug-resistance, migration, invasion in breast cancer and ovarian cancer [19–24]. Besides, PGRN expression in CRC cells was found to promote proliferation and angiogenesis of tumor cells, and correlated with poor disease free survival of patients with CRC in our previous work [25]. Interestingly, local high concentration of PGRN in healing wound could promote proliferation and migration of resident fibroblasts, which could repair and remodel tissue [26], demonstrating the role of PGRN in activating fibroblasts. However, the effect of tumor derived PGRN on the activation of fibroblasts was poorly understood.

Herein, the correlation between PGRN expression in CRC cells and α-SMA expression in fibroblasts in tissues of CRC patients was firstly investigated. Furthermore, the role of PGRN in activating fibroblasts was further evaluated through co-culture assays. We hope our work may provide new insight into the mechanism of PGRN in inducing switch from fibroblast to CAFs.

## RESULTS

### PGRN expression in CRC cells was positively correlated with α-SMA expression in fibroblasts in CRC patient tissues

**Figure 1:**
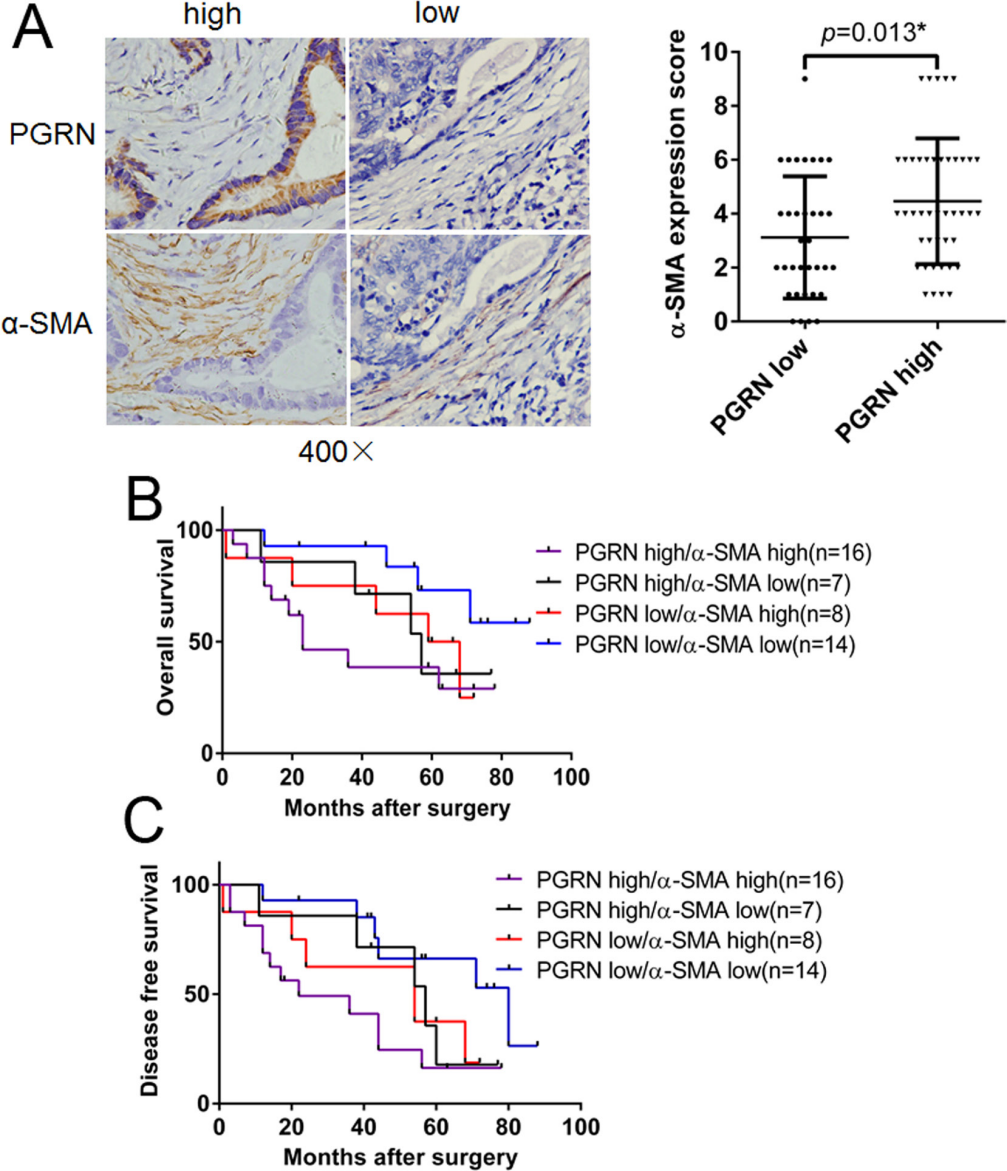
Survival analysis of PGRN and α-SMA expression in CRC tissues (**A**) PGRN and α-SMA expression in CRC tissues were stained using IHC, the difference of α-SMA expression score in tissues with different PGRN expression levels was statistically analyzed. OS (**B**) and DFS (**C**) of patients with different PGRN and α-SMA expression levels were analyzed.

To detect the correlation of PGRN expression in CRC cells and α-SMA in fibroblasts, IHC staining for PGRN and α-SMA was performed in 77 CRC patient tissues. As shown in Figure 1A, α-SMA expression in high PGRN tissue was much stronger than in low PGRN tissue, and their difference was significant indicated by statistical analysis (*p* = 0.013). Then, survival analysis showed that overall survival (OS) and disease free survival (DFS) of CRC patients with both high expression of PGRN and α-SMA were much worse than patients with both low expression of PGRN and α-SMA (*p* = 0.029 and 0.004, respectively; Figure 1B and 1C; Supplementary Tables 1 and 2).

### PGRN derived from CRC cells promoted proliferation, migration and contraction of fibroblasts

To determinate the effect of CRC cells on fibroblast activation, SW480 or SW1116 cells were co-cultured with CCD-18Co cells for 3 days in our cell culture system, and the fibroblasts were separated as described before [27]. Immunocytochemistry and Western blot were used to confirm the purity of CCD-18Co which positive staining with α-SMA, but not E-cadherin (Supplementary Figure 1).

**Figure 2:**
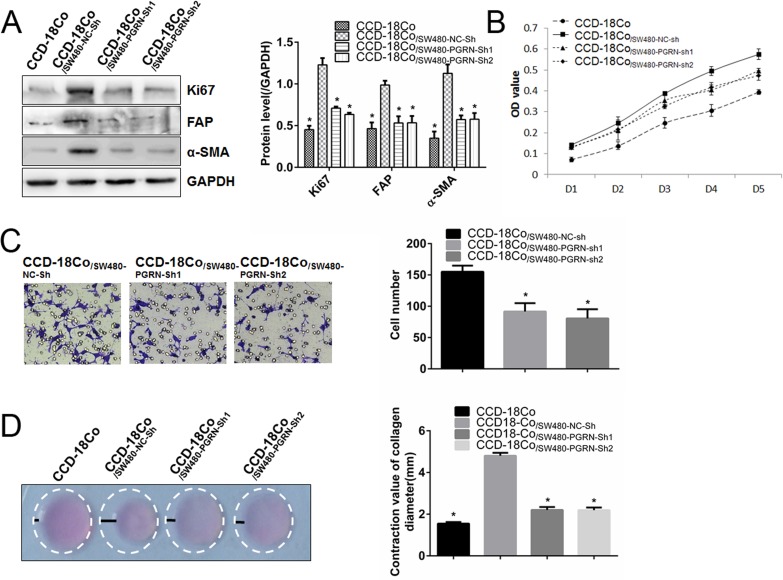
Silencing PGRN expression in SW480 cells inhibited proliferation, migration and contraction abilities of co-cultured fibroblasts After co-culture with SW480-NC-sh, SW480-PGRN-sh1 or SW480-PGRN-sh2 cells, Ki67, FAP and α-SMA protein expression in CCD-18Co cells were calculated (**A**). Ability of proliferation, migration and contraction in co-culture CCD-18Co cells were also evaluated using MTT assay (**B**), Transwellassay (**C**) and gel contraction assay (**D**), respectively. (Magnification 200×) *P < 0.05 was significant.

After co-culture with SW480 cells, Ki67, FAP and α-SMA expression in CCD-18Co cells were up-regulated (Figure 2A). MTT assay and collagen gel contraction assay also showed that both the proliferation (Figure 2B) and contraction (Figure 2D) were enhanced significantly. After co-culture with SW480-PGRN-sh1 or SW480-PGRN-sh2 cells, these promoting effects were abrogated (Figure 2). Consistent with these results, when co-culture with another CRC cell line SW1116-PGRN-sh or SW1116-NC-sh, Ki67, FAP and α-SMA expression were modulated as well (Supplementary Figure 2).

**Figure 3:**
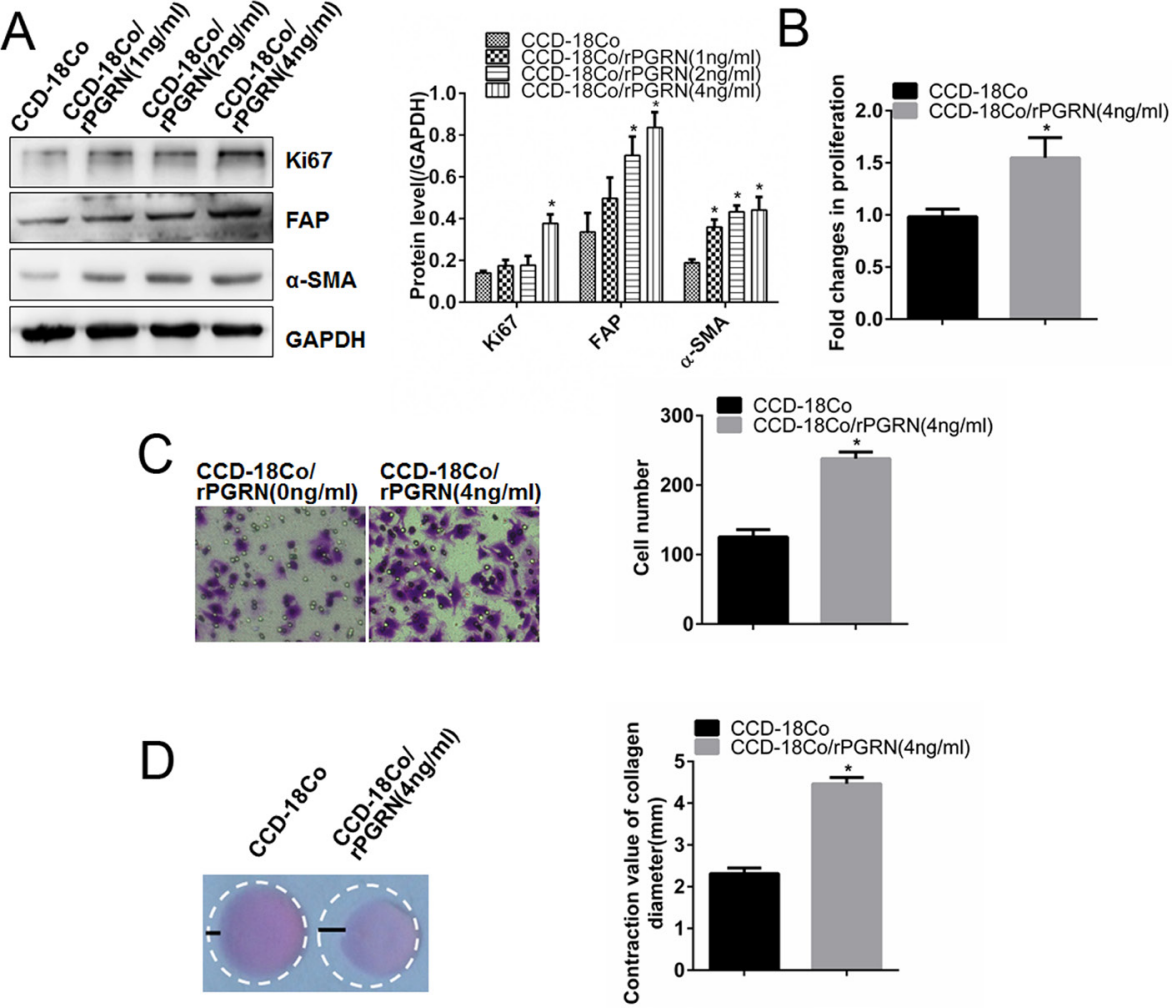
rPGRN promoted proliferation, migration and contraction abilities of fibroblasts After treated with rPGRN, changes of Ki67, FAP and α-SMA expression in CCD-18Co cells were detected using Western blot assay (**A**); changes of proliferation, migration, and contraction abilities of CCD-18Co cells were determined using MTT assay (**B**), Transwell assay (**C**) and gel contraction assay (**D**), respectively. (Magnification 200×) *P < 0.05 was significant.

Moreover, exogenous rPGRN also promoted the expression of Ki67, FAP and α-SMA in CCD-18Co cells (Figure 3A), as well as promoted the proliferation (Figure 3B), migration (Figure 3C) and contraction (Figure 3D) abilities. These results suggested the important role of PGRN derived from CRC cells in activation of fibroblasts.

### AKT and ERK signal pathways were required for regulation of Ki67 and FAP expression in CCD-18Co cells induced by PGRN

**Figure 4:**
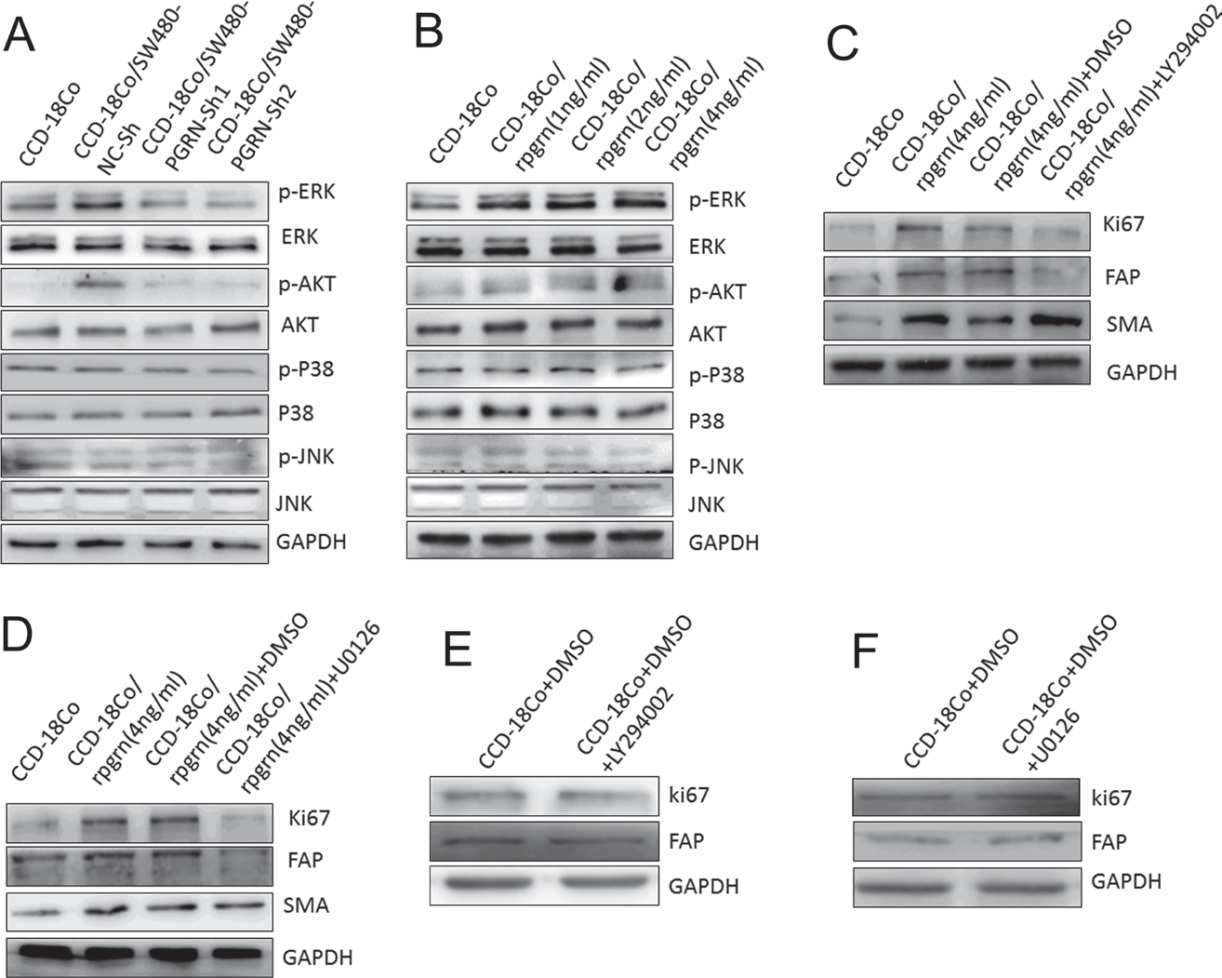
AKT and ERK took part in Ki67 and FAP expression in CCD-18Co cells induced by PGRN After co-culture with SW480-NC-sh/SW480-PGRN-sh cells (**A**) or treated with rPGRN (**B**), phosphorylation of AKT, ERK, P38 and JNK in CCD-18Co cells were detected using Western blot. Treated with LY294002 (**C**) or U0126 (**D**) to block AKT or ERK signal pathway in CCD-18Co cells, Ki67, FAP and α-SMA expression induced by rPGRN were then analyzed using Western blot assay. CCD-18Co cells were also treated by single U0126 (**E**) or LY294002 (**F**).

To identify the potential molecular mechanisms by which PGRN regulates fibroblast activation, several signaling targets were detected. As shown in Figure 4A, after co-culture with SW480 cells, phosphorylation of ERK and AKT in CCD-18Co cells increased significantly but not P38 and JNK. While co-culture with SW480-PGRN-sh1 or SW480-PGRN-sh2 cells, phosphorylation level of ERK and AKT in CCD-18Co cells was particularly lower. rPGRN could also elevate phosphorylation of ERK and AKT in CCD-18Co cells (Figure 4B). Moreover, ERK inhibitor U0126 and AKT inhibitor LY294002 could both abrogate Ki67 and FAP expressions in CCD-18Co cells induced by rPGRN (Figure 4C, 4D). This inhibitory effect on CCD-18Co cells was excluded by U0126 or LY294002 itself (Figure 4E, 4F).

### Tumor necrosis factor receptor 2 (TNFR2) neutralizing antibody could inhibit activation of CCD-18Co cells induced by PGRN partly through AKT signaling pathway

**Figure 5:**
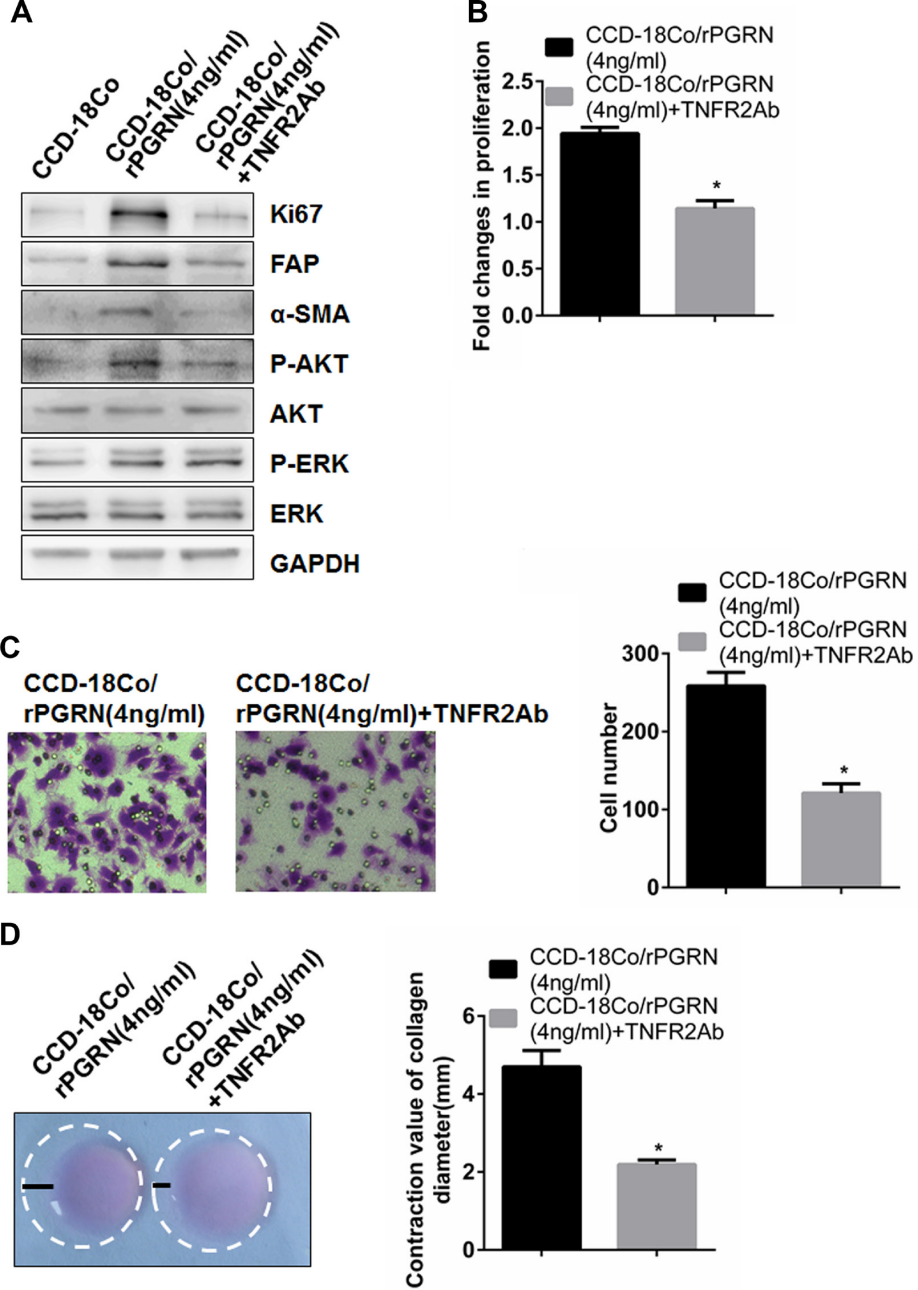
TNFR2 neutralizing antibody inhibited activation and AKT phosphorylation of fibriblasts induced by rPGRN When treated with TNFR2 neutralizing antibody, expression changes of Ki67, FAP, α-SMA, p-AKT and p-ERK in CCD-18Co cells induced by rPGRN were determined using Western blot assay (**A**); abilities of proliferation, migration, and contraction of CCD-18Co cells were also evaluated using MTT assay (**B**), Transwell assay (**C**) and gel contraction assay (**D**), respectively. (Magnification 200×) *P < 0.05 was significant.

It has been reported that PGRN could play important roles in kinds of autoimmune diseases and lung inflammation through binding to TNFR2 [28, 29]. To confirm whether TNFR2 was required for PGRN to activate CCD-18Co cells, we added TNFR2 neutralizing antibody before rPGRN stimulation. The results showed that TNFR2 neutralization antibody significantly inhibited Ki67, FAP and α-SMA expression (Figure 5A). Meanwhile, proliferation (Figure 5B), migration (Figure 5C), and contraction (Figure 5D) of CCD-18Co cells all declined significantly. This suggests that TNFR2 is required for PGRN-mediated activation of CCD-18Co cells.

To further confirm downstream signal pathway through which TNFR2 function in fibroblasts activation, candidate signal targets screened by the above results were detected by western blot assay. As shown in Figure 5A, TNFR2 neutralizing antibody significantly inhibited phosphorylation of AKT stimulated by rPGRN, but no changes of ERK phosphorylation. This suggests that TNFR2 can take part in fibroblast activation partly through AKT signaling pathway.

## DISCUSSION

Tumor progression is a complicated process in which tumor cells interact with tumor microenvironment for mutual promotion. CAFs are considered as a crucial player in tumor microenvironment, which could enhance tumor cell proliferation, invasion, metastasis, and promotion of tumor progression [30–32]. In CRC, the roles of CAFs in invasion of tumor cells and close correlation with poor prognosis of patients have been reported by different groups [33, 34]. As a pro-tumor factor, PGRN was found to play roles in CRC proliferation and angiogenesis in our previous work [25]. But the role of PGRN derived from CRC cells in activating fibroblasts is still unknown.

In this present study, the positive correlation of parenchymal PGRN expression and interstitial α-SMA expression in CRC patient tissues was confirmed. Meanwhile, unfavorable effects of PGRN/α-SMA co-expression on prognosis of CRC patients were also proved for the first time. This reminds us that high levels of PGRN and α-SMA expression could be treated as a marker for predicting prognosis. Moreover, we not only proved the effect of PGRN from CRC cells on activation of fibroblasts through co-culture assay, but also demonstrated the enhancement of fibroblasts activation by exogenous rPGRN. This may imply that CRC cells could affect adjacent fibroblasts activation at least partly through paracrine secretion of PGRN. The changes of some other genes induced by PGRN could also affect fibroblasts activation, which will be investigated in our future studies.

According to the effects of PGRN derived from CRC cells on fibroblasts activation in this study, together with its roles in proliferation of CRC cells and stimulation of human umbilical vein endothelial cells (HUVECs) in our previous works [25], as well as other PGRN related studies in tumors, we proposed that PGRN should be treated as a pleiotropic pro-tumor molecule. It can not only maintain malignant characteristics of tumor cells directly, but also effect biological behaviors of mesenchymal cells in tumor microenvironment significantly, which promote tumor progression indirectly. The therapy by targeting on PGRN would inhibit CRC progression from multiple perspectives and get better outcome.

Deep understanding of potential molecular mechanisms will do favor to finding more effective therapy targets. MacLean J reported that JNK and AKT were implicated in activating phenoconversion of human heart fibroblasts [35]. But, Okada M reported that Endostatin stimulated proliferation and migration of adult rat cardiac fibroblasts through Akt pathway, not ERK, JNK and p38 MAPK [36]. In this present work, we found that both AKT and ERK took part in expression of Ki67 and FAP in CCD-18Co cells regulated by PGRN, but not JNK and p38. This might be attributed to the differences of action protein and tissue origin of fibroblasts. In CRC cells, our previous study also found that PGRN could promote Ki67 and VEGF-A expression via AKT and ERK signaling pathways [25]. The same molecular mechanism in our different studies implies that AKT and ERK might be treated as common downstream targets for the pleiotropia of PGRN in different cellular components of tumors.

Receptor for PGRN is still in debate now. The binding of PGRN and TNFR2 was firstly reported by Tang W in inflammatory arthritis in mice in 2011 [28]. PGRN/TNFR2 has also been found in chondrocytes, macrophages and neutrophile granulocytes [29, 28, 37]. But, whether TNFR2 is required for PGRN in activating fibroblasts is still unknown. In this present study, although direct binding of PGRN and TNFR2 was not detected, we found that TNFR2 neutralizing antibody could effectively abrogate upregulation of Ki67, FAP and α-SMA expression induced by PGRN. Proliferation, migration and contraction abilities of CCD-18Co cells were also significantly inhibited. However, only stimulation of AKT signaling pathway was inhibited, no change happened to ERK pathway. These results suggest that TNFR2 could take part in activation of fibroblasts induced by PGRN, at least partly via AKT signaling pathway.

**Figure 6:**
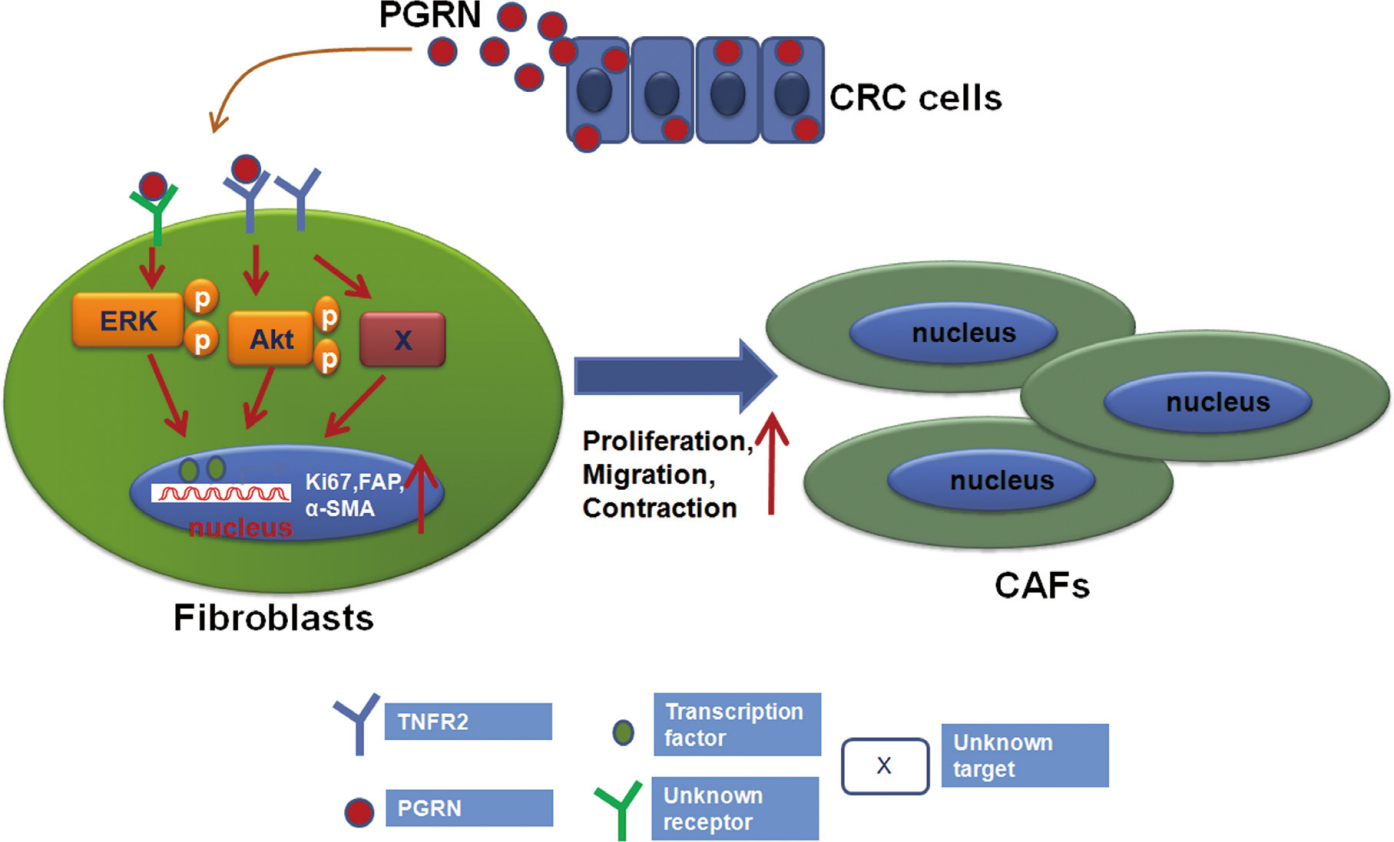
Proposed model for PGRN inducing the fibroblast activation PGRN derived from CRC cells induced the expression of Ki67, FAP and α-SMA molecules partly through TNFR2/AKT and ERK signaling pathways in fibroblasts, stimulated their proliferation, migration and contraction; and ultimately switched fibroblasts to CAFs.

In conclusion, PGRN derived from CRC cells might increase the proliferation, contraction and activation of fibroblasts which were then switched to the CAFs. It was also found that PGRN could induce the expression of Ki67, FAP and α-SMA molecules partly through TNFR2/AKT and ERK signaling pathways, which ultimately leading to the proliferation, migration and contraction of CAFs (Figure 6). These data further emphasize the role of tumor-stroma interactions, in particular fibroblasts, and validate further exploration of the tumor stroma, including PGRN as potential therapeutic target.

## MATERIALS AND METHODS

### Cell lines and cell culture

Human colon interstitial fibroblast cell line CCD-18Co was purchased from American Type Culture Collection (Manassas, VA, USA) and cultured in DMEM medium (Invitrogen, Carlsbad, USA); Colorectal cancer cell lines SW480 and SW1116 were obtained from the Institutes of Biochemistry and Cell Biology (Shanghai, China) which originated from ATCC (Manassas, VA, USA) and cultured in RPMI 1640 medium (Gibco Life Technologies, Grand Island, NY). All culture medium was supplemented with 10% fetal bovine serum (FBS) (Invitrogen, Carlsbad, USA).

### Immunohistochemistry (IHC)

After approval of the Institutional Review Board of Jinan Central Hospital Affiliated to Shandong University, tissue samples from 77 primary colorectal cancer patients were collected from 2006 to 2012. No patients received chemotherapy, radiotherapy or immunotherapy before surgery.

Sections from tumors were cut into 3μm thickness and deparafinized in xylene, hydrated with gradient ethanol. Antigen was repaired and endogenous peroxidase was blocked. Primary antibodies of PGRN (Enzo life science, Farmingdale, NY, USA) and α-SMA (Maixin, Fuzhou, China) were used to incubate the slides followed by incubation with HRP goat anti-rabbit/mouse IgG polymer (Maixin, Fuzhou, China) at room temperature for 20 min, then visualized with DAB (Maixin, Fuzhou, China). Finally, at 400× magnification, scoring process was executed by two investigators at the same time. The percentages of stained tumor cells were divided into four grades (0 = none; 1 ≤ 25%; 26% ≤ 2 ≤ 75%; 3 > 75%). The staining intensity was also divided into four grades (0 meant no staining; 1 meant weak staining; 2 meant intermediate staining; 3 meant strong staining). The expression of PGRN and α-SMA was calculated as the product of proportion score and intensity score (Score ≥ 4 was classified as high expression, < 4 as low expression).

### Transfection

SW480 or SW1116 cells were transfected using HP X-treme GENE HP Reagents (Roche, Basel, Switzerland) by p-GPU6/GFP/Neo/PGRN or control plasmid (Shanghai GenePharmaCo., Ltd, Shanghai, China) to interfere PGRN expression. Cells with silenced PGRN were named as SW480-PGRN-sh or SW1116-PGRN-sh, and control group cells were named as SW480-NC-sh or SW1116-NC-sh. Silencing efficiency of PGRN was detected using RT-PCR, Western blot or ELISA assays (Supplementary Figure 3).

### Co-culture of CRC cells and CCD-18Co cells

2 × 10^5^ SW480 or SW1116 cells were mixed with 1 × 10^5^ CCD-18Co cells and plated in 6-well plate in 2 ml DMEM medium supplemented with 10% FBS for 3 days, then the mixed cells were passaged to a new well in 2ml DMEM medium. After 25 minutes, non-adherent cells in supernatant were discarded and the well was washed using PBS gently for 3 times [27], cells still adhering to bottom of the well were considered as fibroblasts and named as CCD-18Co_/SW480_ or CCD-18Co_/SW1116_ cells. The purity was established by detecting E-cadherin and α-SMA expression using Immunocytochemistry or Western blot assay. The separated fibroblasts were calculated and further used for detecting ki-67, FAP, and α-SMA expression using the following Immunocytochemistry, Reverse Transcriptase-Polymerase Chain Reaction (RT-PCR) and Western Blot assays; and determining the abilities of proliferation, migration and contraction through MTT assay, migration assay, and collagen gel contraction assay, respectively.

### Immunocytochemistry

Fibroblasts were pre-plated on the cover glass in 6-well plate. 24 hours later, cells were fixed with methanol, blocked with H2O2 and goat serum. Then, cells were incubated using primary antibodies of E-cadherin (Cell Signaling Technology, Danvers, MA, USA) or α-SMA (Maixin, Fuzhou, China) at 4°C overnight and second antibody for 20 min at room temperature, followed by DAB and hematoxylin staining.

### Reverse transcriptase-polymerase chain reaction (RT-PCR)

Cells were cleaved by Trizol (Invitrogen, Carlsbad, CA, USA) and RNA was extracted following the standard protocols. PrimeScript RT-PCR Kit (TaKaRa, China) was used for reverse transcription to get cDNA. At last, polymerase chain reaction was executed to get objective fragments. Primers were as follows: PGRN: F 5′-ATCTTTACCGTCTCAGGGACTT-3′ R 5′- CCATCGACCATAACACAGCAC-3′; GAPDH: F 5′-A GAAGGCTGGGGCTCATTTG-3′R 5′-AGGGGCCATCC ACAGTCTTC-3′.

### Western blot assay

Equal protein from CCD-18Co (separated from different co-culture system) was added into the roles of SDS-PAGE. After separation, protein was transferred to PVDF membrane (Millipore, USA). The primary antibodies (Supplementary Table 3) and secondary antibodies (Proteintech Group, China) were used to probe protein on the membrane. Then the antigen-antibody compound was visualized by chemiluminescence solution (Millipore Corporation, Billerica, MA, USA), the band intensity was measured by image J software.

To determine whether AKT and ERK signal pathways were required for regulation of Ki67 and FAP expression, CCD-18Co cells were pre-plated in 6-well plate at 2 × 10^5^ cells per well for 24 hours, then the medium was replaced by DMEM medium without FBS containing different concentrations of rPGRN (0, 4 ng/ml), ERK inhibitor U0126 (4 ng/ml, diluted with DMSO) and AKT inhibitor LY294002 (4 ng/ml, diluted with DMSO). 48 hours later, western blot assay was performed, and Ki67 and FAP expression was calculated.

### Cell proliferation assay

MTT (Beijing solar bio science & technology co. ltd., China) assay was used to detect cell proliferation ability. After co-culture with SW480 transfected with PGRN shRNA or control plasmid, fibroblasts were separated and plated in 96-well plate at 1500 cells per well in triplicate. Cell viability was measured at 24, 48, 72, 96, and 120 hours.

To detect the effects of human recombinant PGRN (rPGRN) (Sino Biological Inc., Beijing, China; cat NO. 10826-H08H) on proliferation ability, CCD-18Co cells were pre-plated in 96-well plate at 2000 cells per well in triplicate for 24 hours, then the medium was replaced by DMEM medium without FBS containing different concentrations of rPGRN (0, 4 ng/ml). 48 hours later, MTT assay was performed and OD value was calculated.

To confirm whether TNFR2 was required for PGRN to activate CCD-18Co cell proliferation, TNFR2 neutralizing antibody (2 ug/ml) (R&D Systems; cat NO. MAB726–100) was added to CCD-18Co cell culture (2000 cells per well in 96-well plate) for 24 hour before rPGRN stimulation.

### Cell migration assay

Boyden chamber (BD Biosciences, Bedford, MA, USA) was used to detect cell migration ability. To detect effect of PGRN derived from SW480 cells on migration of fibroblasts, CCD-18Co cells were plated in the upper chambers in DMEM without FBS, conditioned medium (CM) from SW480 cells transfected with PGRN shRNA or control plasmid was plated in the lower chamber. 6 hours later, cells that moved to the lower surface of polycarbonate membrane were stained with 0.1% crystal violet and counted at five random 200× fields to get the sum.

To detect the effects of rPGRN on migration of fibroblasts, CCD-18Co cells pre-treated with different concentrations of rPGRN (0, 4 ng/ml) for 48h were plated in the upper chambers in DMEM without FBS, DMEM supplemented with 5% FBS was plated in the lower chamber. 2 hours later, cells that moved to the lower surface of polycarbonate membrane were stained with 0.1% crystal violet and counted at five random 200× fields to get the sum.

When to determine the TNFR2 effect, TNFR2 neutralizing antibody (2ug/ml) was added to CCD-18Co cells for 24 hour before rPGRN stimulation. Then the process was same as the upper assay.

### Collagen gel contraction assay

350 μl DMEM medium with 5 × 10^5^/ml fibroblasts supplemented with 10% FBS with rPGRN (4 ng/ml) were mixed with 150 μl rat tail glue (Beijing solarbio science & technology co. ltd., Beijing, China), then the mixture was plated in 24-well plate and placed to polymerize in 37°C for 30 min, 500 μl medium was added into the well and the gel was released from well wall using pipette tip. 48 hours later, the diameters of gel were measured and normalized to that of gel at beginning.

### Statistical analysis

SPSS 11.0 software was used and data were expressed as Means ± SD, the difference between two groups was analyzed using student's *t* test. Kaplan-Meier method was used to draw survival curves and log-rank test was used to compare the difference. *P* < 0.05 was considered significant.

## SUPPLEMENTARY MATERIALS FIGURES AND TABLES



## References

[1] Shih YH, Peng CL, Lee SY, Chiang PF, Yao CJ, Lin WJ, Luo TY, Shieh MJ (2015). 111In-cetuximab as a diagnostic agent by accessible epidermal growth factor (EGF) receptor targeting in human metastatic colorectal carcinoma. Oncotarget.

[2] Rui Y, Wang C, Zhou Z, Zhong X, Yu Y (2015). K-Ras mutation and prognosis of colorectal cancer: a meta-analysis. Hepato-gastroenterology.

[3] Yun HM, Park KR, Kim EC, Han SB, Yoon DY, Hong JT (2015). IL-32αsuppresses colorectal cancer development via TNFR1 mediated death signaling. Oncotarget.

[4] Trylcova J, Busek P, Smetana K, Balaziova E, Dvorankova B, Mifkova A, Sedo A (2015). Effect of cancer-associated fibroblasts on the migration of glioma cells *in vitro*. Tumor Biol.

[5] Marsh T, Pietras K, McAllister SS (2013). Fibroblasts as architects of cancer pathogenesis. Biochim Biophys Acta.

[6] Martinez-Outschoorn UE, Trimmer C, Lin Z, Whitaker-Menezes D, Chiavarina B, Zhou J, Wang C, Pavlides S, Martinez-Cantarin MP, Capozza F, Witkiewicz AK, Flomenberg N, Howell A (2014). Autophagy in cancer associated fibroblasts promotes tumor cell survival. Cell Cycle.

[7] Zheng SG, Jia CC, Wang TT, Liu W, Fu BS, Hua X, Wang GY, Li TJ, Li X, Wu XY, Tai Y, Zhou J, Chen GH (2013). Cancer-Associated Fibroblasts from Hepatocellular Carcinoma Promote Malignant Cell Proliferation by HGF Secretion. PLoS ONE.

[8] Zhou Z, Taguchi A, Kawana K, Tomio K, Yamashita A, Isobe Y, Nagasaka K, Koga K, Inoue T, Nishida H, Kojima S, Adachi K, Matsumoto Y (2014). Matrix Metalloproteinase (MMP)-9 in Cancer-Associated Fibroblasts (CAFs) Is Suppressed by Omega-3 Polyunsaturated Fatty Acids *In Vitro* and *In Vivo*. PLoS ONE.

[9] Yu Y, Xiao CH, Tan LD, Wang QS, Li XQ, Feng YM (2013). Cancer-associated fibroblasts induce epithelial–mesenchymal transition of breast cancer cells through paracrine TGF-β signalling. Br J Cancer.

[10] Horimoto Y, Polanska UM, Takahashi Y, Orimo A (2014). Emerging roles of the tumor-associated stroma in promoting tumor metastasis. Cell Adh Migr.

[11] Cirri P, Chiarugi P (2012). Cancer-associated-fibroblasts and tumour cells: a diabolic liaison driving cancer progression. Cancer Metastasis Rev.

[12] Xouri G, Christian S (2010). Origin and function of tumor stroma fibroblasts. Semin Cell Dev Biol.

[13] Rasanen K, Vaheri A (2010). Activation of fibroblasts in cancer stroma. Exp Cell Res.

[14] Bateman A, Bennett HP (2009). The granulin gene family: from cancer to dementia. Bioessays.

[15] Liu CJ, Bosch X Progranulin: a growth factor, a novel TNFR ligand and a drug target. Pharmacol Ther.

[16] Zhu J, Nathan C, Jin W, Sim D, Ashcroft GS, Wahl SM, Lacomis L, Erdjument-Bromage H, Tempst P, Wright CD, Ding A (2002). Conversion of proepithelin to epithelins: roles of SLPI and elastase in host defense and wound repair. Cell.

[17] Suzuki M, Nishiahara M (2002). Granulin precursor gene: a sex steroid-inducible gene involved in sexual differentiation of the rat brain. Mol Genet Metab.

[18] De Muynck L, Van Damme P (2011). Cellular effects of progranulin in health and disease. J Mol Neurosci.

[19] Abrhale T, Brodie A, Sabnis G, Macedo L, Tian C, Yue B, Serrero G (2011). GP88 (PC-Cell Derived Growth Factor, progranulin) stimulates proliferation and confers letrozole resistance to aromatase overexpressing breast cancer cells. BMC Cancer.

[20] Diaz-Cueto L, Arechavaleta-Velasco F, Diaz-Arizaga A, Dominguez-Lopez P, Robles-Flores M (2012). PKC signaling is involved in the regulation of progranulin (acrogranin/PC-cell-derived growth factor/granulin-epithelin precursor) protein expression in human ovarian cancer cell lines. Int J Gynecol Cancer.

[21] Han JJ, Yu M, Houston N, Steinberg SM, Kohn EC (2011). Progranulin is a potential prognostic biomarker in advanced epithelial ovarian cancers. Gynecol Oncol.

[22] Wang M, Li G, Yin J, Lin T, Zhang J (2012). Progranulin overexpression predicts overall survival in patients with glioblastoma. Med Oncol (Northwood, London, England).

[23] Cheung ST, Cheung PF, Cheng CK, Wong NC, Fan ST (2011). Granulin-epithelin precursor and ATP-dependent binding cassette (ABC)B5 regulate liver cancer cell chemoresistance. Gastroenterology.

[24] Frampton G, Invernizzi P, Bernuzzi F, Pae HY, Quinn M, Horvat D, Galindo C, Huang L, McMillin M, Cooper B, Rimassa L, DeMorrow S (2012). Interleukin-6-driven progranulin expression increases cholangiocarcinoma growth by an Akt-dependent mechanism. Gut.

[25] Yang D, Wang LL, Dong TT, Shen YH, Guo XS, Liu CY, Liu J, Zhang P, Li J, Sun YP (2015). Progranulin promotes colorectal cancer proliferation and angiogenesis through TNFR2 Akt and ERK signaling pathways. Am J Cancer Res.

[26] He Z, Ong CH, Halper J, Bateman A (2003). Progranulin is a mediator of the wound response. Nat Med.

[27] Zhang CY, Fu L, Fu JH, Hu L, Yang H, Rong TH, Li Y, Liu HB, Fu SB, Zeng YX, Guan XY (2009). Fibroblast growth factor receptor 2-positive fibroblasts provide a suitable microenvironment for tumor development and progression in esophageal carcinoma. Clin Cancer Res.

[28] Tang W, Lu Y, Tian QY, Zhang Y, Guo FJ, Liu G, Syed NM, Lai Y, Lin EA, Kong L, Su J, Yin F, Ding AH (2011). The growth factor progranulin binds to TNF receptors and is therapeutic against inflammatory arthritis in mice. Science.

[29] Guo Z, Li Q, Han Y, Liang Y, Xu Z, Ren T (2012). Prevention of LPS-induced acute lung injury in mice by progranulin. Mediators Inflamm.

[30] Tlsty TD (2001). Stromal cells can contribute oncogenic signals. Semin Cancer Biol.

[31] Micke P, Ostman A (2004). Tumour-stroma interaction: cancer-associated fibroblasts as novel targets in anti-cancer therapy?. Lung Cancer.

[32] Orimo A, Weinberg RA (2006). Stromal fibroblasts in cancer: a novel tumor-promoting cell type. Cell Cycle.

[33] De Wever O, Nguyen QD, Van Hoorde L, Bracke M, Bruyneel E, Gespach C, Mareel M (2004). Tenascin-C SF/HGF produced by myofibroblasts *in vitro* provide convergent proinvasive signals to human colon cancer cells through RhoA, Rac. FASEB J.

[34] Herrera M, Islam ABMMK, Herrera A, Martin P, Garcia V, Silva J, Garcia JM, Salas C, Casal I, de Herreros AG, Bonilla F, Pena C (2013). Functional Heterogeneity of Cancer-Associated Fibroblasts from Human Colon Tumors Shows Specific Prognostic Gene Expression Signature. Clin Cancer Res.

[35] MacLean J, Pasumarthi KB (2014). Signaling mechanisms regulating fibroblast activation, phenoconversion and fibrosis in the heart. Indian J Biochem Biophys.

[36] Okada M, Oba Y, Yamawaki H (2015). Endostatin stimulates proliferation and migration of adult rat cardiac fibroblasts through PI3K/Akt pathway. Eur J Pharmacol.

[37] Jian J, Zhao S, Tian QY, Gonzalez-Gugel E, Mundra JJ, Uddin SM, Liu B, Richbourgh B, Brunetti R, Liu CJ (2013). Progranulin directly binds to the CRD2 and CRD3 of TNFR extracellular domains. FEBS Lett.

