# Supplementary material for: Tumor necrosis factor receptor 2/AKT and ERK signaling pathways contribute to the switch from fibroblasts to CAFs by progranulin in microenvironment of colorectal cancer
Source: Oncotarget. 2017 Feb 17;8(16):26323–33. doi: 10.18632/oncotarget.15461 (PMC5432260; doi:10.18632/oncotarget.15461)
Supplement: Supplementary file 1 [file oncotarget-08-26323-s001.pdf]

Tumor necrosis factor receptor 2/AKT and ERK signaling pathways contribute to the switch from fibroblasts to CAFs by progranulin in microenvironment of colorectal cancer

Supplementary Materials

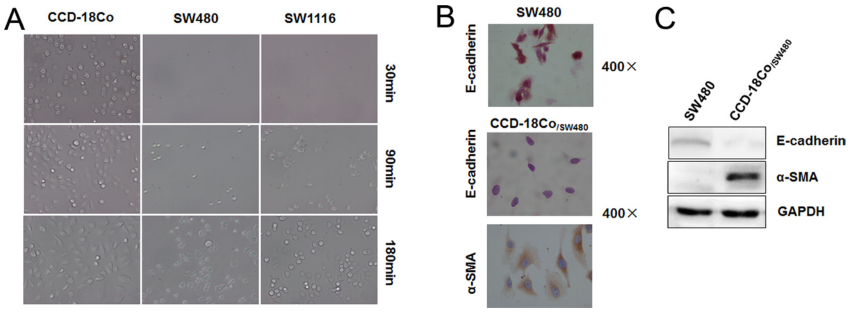

Supplementary Figure 1: Isolation and identification of fibroblasts after co-cultured with CRC cells. (**A**) Adhesion status of CCD-18Co cells, SW480 and SW1116 cells at 30 min, 90 min and 180 min (Magnification 200×). (**B**) E-cadherin expression of SW480 cells, E-cadherin and α-SMA expression of CCD-18Co$_{/SW480}$ cells were detected using immunohistochemistry (Magnification 400×). (**C**) E-cadherin and α-SMA expression of SW480 cells and CCD-18Co$_{/SW480}$ cells were detected using Western blot assay.

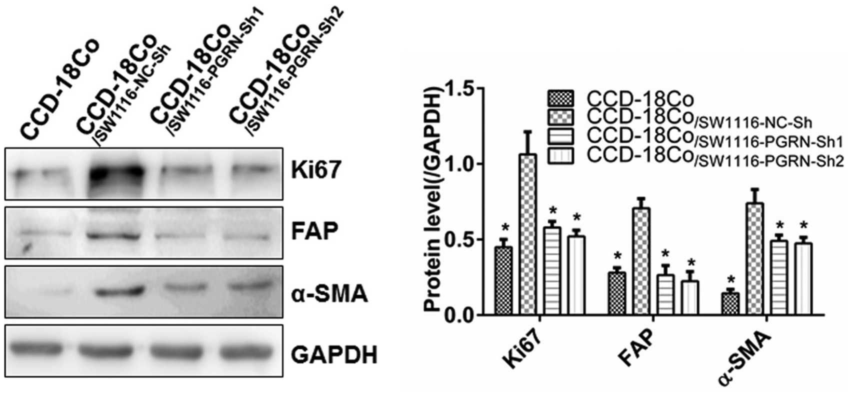

Supplementary Figure 2: Silencing PGRN in SW1116 cells inhibited Ki67, FAP and α-SMA expression of co-cultured fibroblasts. After co-culture with SW1116-NC-sh, SW1116-PGRN-sh1 or SW1116-PGRN-sh2 cells, Ki67, FAP and α-SMA protein expression in CCD-18Co cells were calculated. $^*P < 0.05$ was significant.

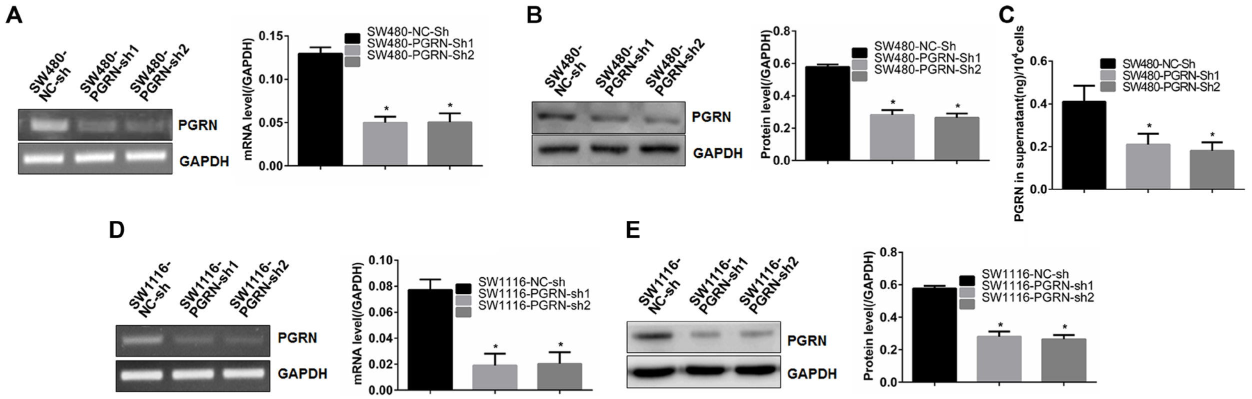

Supplementary Figure 3: PGRN expression in CRC cells after transfection. After transfection, PGRN expression in SW480 cells was detected using RT-PCR (**A**), Western blot (**B**) and ELISA assays (**C**). PGRN expression in SW1116 cells was also confirmed using RT-PCR (**D**) and Western blot (**E**). **P* < 0.05 was significant.

Supplementary Table 1: OS analysis of patients with different expression levels of PGRN and α-SMA

OS	*P*
PGRN(high)/α-SMA(high) VS PGRN(high)/ α-SMA(low)	0.458
PGRN(high)/ α-SMA(high) VS PGRN(low)/ α-SMA(high)	0.531
PGRN(high)/ α-SMA(low) VS PGRN(low)/ α-SMA(high)	0.873
PGRN(high)/ α-SMA(low) VS PGRN(low)/ α-SMA(low)	0.192
PGRN(low)/ α-SMA(high) VS PGRN(low)/ α-SMA(low)	0.174
PGRN(high)/ α-SMA(high) VS PGRN(low)/ α-SMA(low)	0.029*

*P <0.05 was significant

Supplementary Table 2: DFS analysis of patients with different expression levels of PGRN and α-SMA

DFS	*P*
PGRN(high)/ α-SMA(high) VS PGRN(high)/ α-SMA(low)	0.267
PGRN(high)/ α-SMA(high) VS PGRN(low)/ α-SMA(high)	0.311
PGRN(high)/ α-SMA(low) VS PGRN(low)/ α-SMA(high)	0.98
PGRN(high)/ α-SMA(low) VS PGRN(low)/ α-SMA(low)	0.102
PGRN(low)/ α-SMA(high) VS PGRN(low)/ α-SMA(low)	0.077
PGRN(high)/ α-SMA(high) VS PGRN(low)/ α-SMA(low)	0.004*

*$P < 0.05$ was significant.

Supplementary Table 3: Primary antibodies used in Western blot assay

Name	Dilution ratio	Company
PGRN	1:400	Enzo life science, Farmingdale, NY,USA
Ki67	1: 800	abcam, Cambridge, MA,USA
FAP	1:1000	Proteintech Group Inc., Chicago, USA
E-cadherin	1:1000	Cell Signaling Technology, Danvers, MA, USA
α-SMA	1:800	abcam, Cambridge, MA,USA
phospho-ERK1/2	1:1000	Epitomics, Burlingame, CA, USA
ERK1/2	1:1000	Cell Signaling Technology, Danvers, MA, USA
phospho-Akt	1:1000	Cell Signaling Technology, Danvers, MA, USA
Akt	1:1000	Cell Signaling Technology, Danvers, MA, USA
phospho-P38	1:1000	Cell Signaling Technology, Danvers, MA, USA
P38	1:1000	Cell Signaling Technology, Danvers, MA, USA
phospho-JNK	1:1000	Epitomics, Burlingame, CA, USA
JNK	1:1000	Cell Signaling Technology, Danvers, MA, USA
GAPDH	1:5000	Proteintech Group, Inc., Wuhan, China
